# Super-resolution optical microscopy using cylindrical vector beams

**DOI:** 10.1515/nanoph-2022-0241

**Published:** 2022-06-27

**Authors:** Min Liu, Yunze Lei, Lan Yu, Xiang Fang, Ying Ma, Lixin Liu, Juanjuan Zheng, Peng Gao

**Affiliations:** School of Physics, Xidian University, Xi’an 710071, China; Guangzhou Institute of Technology, Xidian University, Guangzhou 510555, China; School of Optoelectronic Engineering, Xidian University, Xi’an 710071, China

**Keywords:** cylindrical vector beam, far-field microscopy, near-field microscopy, super-resolution microscopy

## Abstract

Super-resolution optical microscopy, which gives access to finer details of objects, is highly desired for fields of nanomaterial, nanobiology, nanophotonics, etc. Many efforts, including tip optimization and illumination optimization etc., have been made in both near-field and far-field super-resolution microscopy to achieve a spatial resolution beyond the diffraction limit. The development of vector light fields opens up a new avenue for super-resolution optical microscopy via special illumination modes. Cylindrical vector beam (CVB) has been verified to enable resolution improvement in tip-scanning imaging, nonlinear imaging, stimulated emission depletion (STED) microscopy, subtraction imaging, superoscillation imaging, etc. This paper reviews recent advances in CVB-based super-resolution imaging. We start with an introduction of the fundamentals and properties of CVB. Next, strategies for CVB based super-resolution imaging are discussed, which are mainly implemented by tight focusing, depletion effect, plasmonic nanofocusing, and polarization matching. Then, the roadmap of super-resolution imaging with CVB illumination in the past two decades is summarized. The typical CVB-based imaging techniques in fields of both near-field and far-field microscopy are introduced, including tip-scanning imaging, nonlinear imaging, STED, subtraction imaging, and superoscillation imaging. Finally, challenges and future directions of CVB-illuminated super-resolution imaging techniques are discussed.

## 1 Introduction

Optical microscopy has provided a powerful tool for understanding the nature and rule of matter due to its ability of indicating microscopic details [1, 2]. In recent years, optical microscopy with resolution beyond the optical diffraction limit has been widely demanded in fields of materials [3], chemistry [4], biology [5], etc. Super-resolution optical microscopy including near-field microscopy (typically tip enhanced Raman spectroscopy, TERS) [6], [7], [8] and far-field microscopy (typically localization microscopy [9], [10], [11]; structured illumination microscopy, SIM [12], [13], [14]; stimulated emission depletion microscopy, STED [15], [16], [17]) with nanoscale resolution has broken the diffraction limit, which has significantly accelerated the advancement of nanoscience. Great efforts have been made to improve the spatial resolution [18], as well as the image contrast [19], field-of-view [20], wavelength range [21], compatibility [22], etc.

Near-field microscopy detects near-field evanescent waves and gives access to nanoscale information at a single molecule level [23], [24], [25]. The common approach for higher resolution in near-field microscopy is tip optimization, including shape optimization [26], tip direction adjustment [27], chemical component change [28], [29], [30], nanograting modification [31] etc. Far-field super-resolution microscopic approaches can reach a spatial resolution of ∼10 nm [32] by innovating the illumination methods to tune fluorescence molecules on and off, and has irreplaceable advantages of biocompatibility [33], fast imaging speed [34], and wide field-of-view [35].

With the development of vector light field, vectorial illumination, and notably cylindrical vector illumination, opens up new avenues for both near-field and far-field super-resolution microscopy. Radially polarized vector beams (RVBs) and azimuthally polarized vector beams (AVBs), which are the most widely-used cylindrical vector beams (CVBs), have contributed a lot to resolution improvement. In contrast to the conventional linearly polarized beam that has Gaussian energy distribution and a certain polarization direction on the beam cross-section, CVBs have doughnut-like

(1) $$\begin{array}{ll} E_{\text{RVB}}\left(r,z\right) & =-E_{0}J_{1}\left(\frac{\beta r}{1+iz/z_{0}}\right)\exp\left[-\frac{i\beta^{2}z/\left(2k\right)}{1+iz/z_{0}}\right]\\ & \;\cdot u\left(r,z\right)\exp\left[i\left(kz-\omega t\right)\right]e_{r} \end{array}$$

(2) $$\begin{array}{ll} E_{\text{AVB}}\left(r,z\right) & =E_{0}J_{1}\left(\frac{\beta r}{1+iz/z_{0}}\right)\exp\left[-\frac{i\beta^{2}z/\left(2k\right)}{1+iz/z_{0}}\right]\\ & \;\cdot u\left(r,z\right)\exp\left[i\left(kz-\omega t\right)\right]e_{\phi } \end{array}$$

energy distribution, radial/azimuthal polarization distribution, and strong longitudinal/transverse focusing field component [36]. Hence, CVBs can be further used as tightly focused illumination sources in near-field microscopy or depletion beams of STED for far-field microscopy. In near-field microscopy, the radial-polarization light field can realize plasmonic nanofocusing on a metallized fiber tip [37], [38], [39], [40], [41], yielding considerable signal enhancement [42, 43] and ultrahigh resolution for tip-scanning imaging [44]. In far-field microscopy, RVBs and AVBs have been reported to be applied as pump beams [45, 46] and depletion beams [47] in STED microscopy for enhanced resolution in lateral and axial. Besides, CVBs have also expanded their applications in nonlinear microscopy [48], subtraction microscopy [49], superoscillation microscopy [50], etc.

This paper reviews the recent progresses in CVB-assisted super-resolution imaging approaches. We start with the fundamentals and properties of CVBs. Next, strategies for CVB-assisted super-resolution imaging and roadmap of the past-two-decade development of CVB-based super-resolution microscopic approaches are summarized. Then, the typical CVB-based super-resolution imaging techniques in fields of both near-field and far-field microscopy are introduced. Finally, challenges and future directions of CVB-illuminated super-resolution imaging techniques are discussed.

## 2 CVB: fundamentals and properties

CVBs generally have doughnut-shaped intensity distribution on the cross-section of a light beam, which is dark at the center and brilliant all around. Note that CVBs throughout the whole text indicate RVBs and AVBs, while other doughnut-shaped vector beams are not considered. Unlike RVB and AVB, the commonly-used Gaussian beam is a scalar beam, which is the mathematical solution of the scalar Helmholtz equation in the paraxial limit. However, the vector light field is a vector solution of Maxwell’s vector wave equation [51], which possesses special polarization characteristics that the polarization states are different at different position of the beam cross-section. Both RVBs and AVBs have axisymmetric polarization distributions [52], [53], [54]. RVB is divided with AVB for different polarization distributions on the beam cross-section, the former is radial [55], [56], [57], and the latter is azimuthal [58], [59], [60].

**Figure 1:**
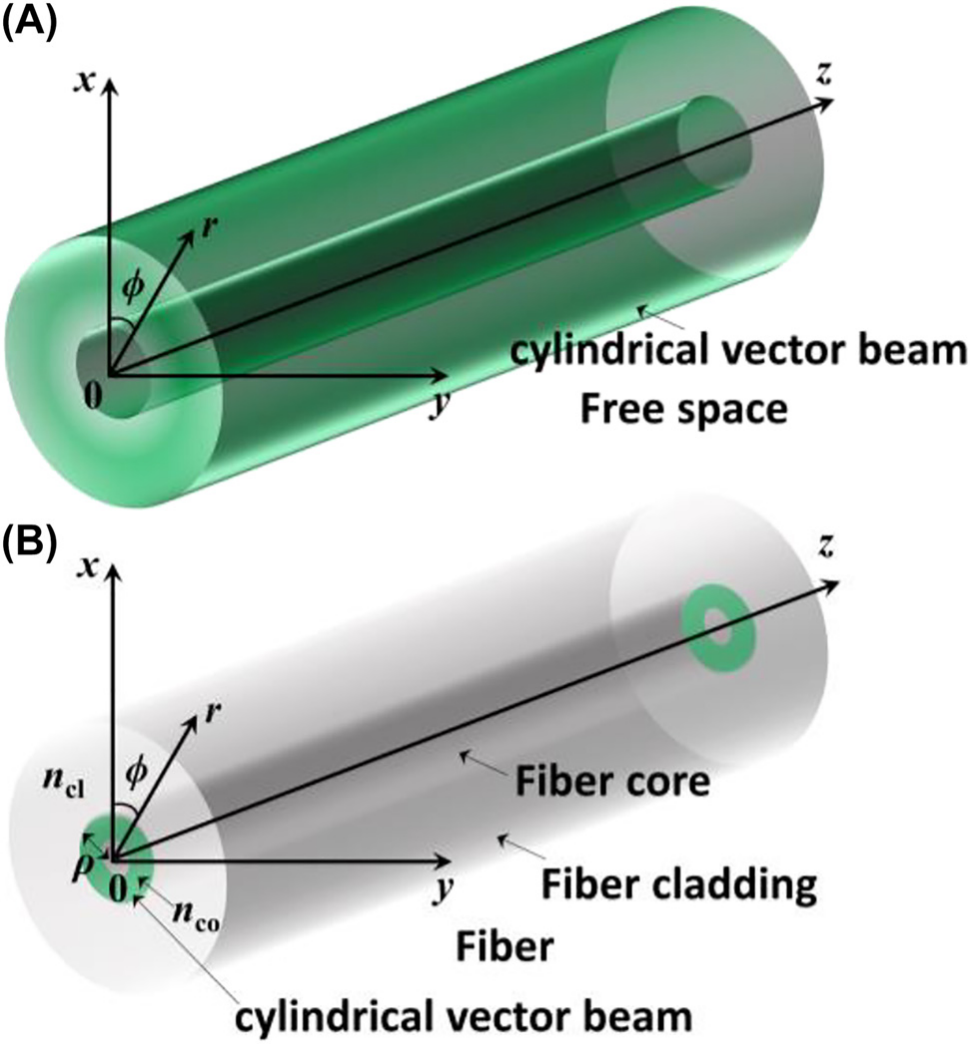
Diagrams of CVBs. (A) Diagram of CVB in free space. (B) Diagram of CVB in fiber.

Figure 1 shows diagrams of CVBs in free space and in fiber. Corresponding to the coordinates exhibited in Figure 1A, the mathematical expressions of RVB and AVB in free space are respectively listed in Eqs. (1) and (2) [51, 52], where *E*_0_ is a constant electric-field amplitude; *J*_1_(*x*) is the first-order Bessel function of the first kind; *β* is a constant scale parameter; *u*(*r*, *z*) is the fundamental Gaussian solution; ***e***_***r***_ is the unit vector in the radial direction; ***e***_***ϕ***_ is the unit vector in the azimuthal direction. Note that Eqs. (1) and (2) refer to typical vector Bessel–Gaussian beams, apart from which there also exist Laguerre–Gaussian typed trial-solutions [61], which are not listed here, corresponding to vector Laguerre–Gaussian beams. Both Bessel–Gaussian and Laguerre–Gaussian-typed solutions indicate the axial-symmetry characteristics and radial or azimuthal polarization distribution on the beam cross-section.

Originated from the boundary conditions of fiber, Maxwell’s vector wave equations have different solutions in fiber from that in free space, which leads to different mathematical expressions of RVB and AVB in fiber compared with that in free space. Corresponding to the coordinates shown in Figure 1B, the in-fiber RVB and AVB, respectively, obey the intrinsic equations below [62]:

(3) $$\frac{n_{\text{co}}^{2}J_{1}\left(U\right)}{UJ_{0}\left(U\right)}+\frac{n_{\text{cl}}^{2}K_{1}\left(W\right)}{WK_{0}\left(W\right)}=0$$

(4) $$\frac{J_{1}\left(U\right)}{UJ_{0}\left(U\right)}+\frac{K_{1}\left(W\right)}{WK_{0}\left(W\right)}=0$$

where *n*_co_ and *n*_cl_ are the refractive index of fiber core and fiber cladding, respectively; *J*_1_(*x*) and *J*_0_(*x*) are the first-order and zero-order Bessel function of the first kind, respectively; *K*_1_(*x*) and *K*_0_(*x*) are the first-order- and zero-order-modified Bessel function of the first kind, respectively; *U* and *W* are the dimensionless modal parameters for the core and cladding, the mathematical expressions of which are listed below:

(5) $$U_{j}=\rho {\left(k^{2}n_{\text{co}}^{2}-\beta_{j}^{2}\right)}^{1/2}$$

(6) $$W_{j}=\rho {\left(\beta_{j}^{2}-k^{2}n_{\text{cl}}^{2}\right)}^{1/2}$$

where *ρ* is the core radius of fiber; *β*_*j*_ is the propagation constant.

RVB and AVB have similar energy distribution but different polarization characteristics on the cross-section of the light beam, no matter they are in free space or in fiber. The energy distribution obeys the doughnut-shape distribution on the beam cross-section, the reason of which is that both RVB and AVB have undefined polarization at the center of the beam cross-section due to the singularity, which results in central dark spot.

**Figure 2:**
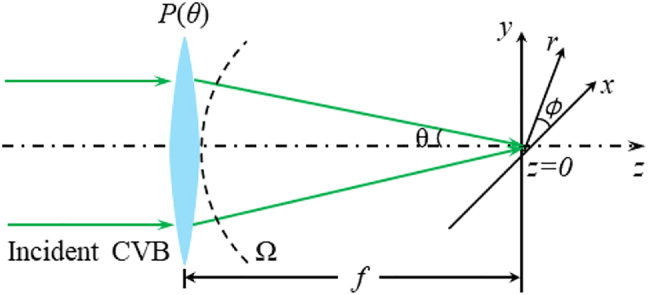
Sketch map of the focusing process of RVB.

In addition to the optical properties of energy and polarization on the cross-section of the light beam, the focusing characteristics have also achieved much attention. For free space, it is theoretically verified in 2000 by Quabis et al. [63] and Youngworth and Brown [64] that a large longitudinally polarized-field component can be obtained via RVB under tightly focusing condition. Figure 2 depicts the focusing process of CVB in free space. The focal length of the lens is *f*, and *z* equals 0 in the focal plane. The mathematical expression of the focal field near the focal plane is listed in Eq. (7).

(7) $$E\left(r,\phi ,z\right)=E_{r}e_{r}+E_{\phi }e_{\phi }+E_{z}e_{z}$$

where ***e***_***r***_ *,* ***e***_***ϕ***_ and ***e***_***z***_, respectively, refer to the unit vectors along the radial, azimuthal, and longitudinal directions. *E*_*r*_, *E*_*φ*_, and *E*_*z*_ are the corresponding amplitudes, which can be can be written out based on Richards-Wolf vectorial diffraction method [52, 65, 66]. *E*_*r*_, *E*_*ϕ*_, and *E*_*z*_ can be, respectively, expressed by Eqs. (8)–(10) for RVB and (11)–(13) for AVB [64], where *J*_1_(*x*) is the first-order Bessel function of the first kind; *J*_0_(*x*) is the zero-order Bessel function of the first kind; *P*(*θ*) refers to the pupil plane apodization function. Different objective lenses may have different *P*(*θ*) due to the different ray projection functions that obey sine condition [67], Herschel condition [68], or Lagrange condition [69], etc., where the most commonly used type is the sine condition. It can be seen that the focal field of RVB still possesses cylindrical symmetry. Moreover, Eq. (10) reveals that the *z* component, which is also called longitudinal component, is significantly strong attributed to the *J*_0_ Bessel function. However, the focal field of AVB has a purely transverse polarization.

Besides the theoretical researches on focusing characteristics of RVB mentioned above, Dorn et al. experimentally realized RVB focusing with a size of 0.16*λ* ^2^, which is significantly smaller than that for a linearly polarized beam (LPB) [36]. A parabolic mirror with high NA has also been used to focus RVB into a diffraction-limited spot at visible lights [70, 71], or even applied in TERS to overcome the problem of NA limitations brought by the conventional geometry [72]. Lately, the development of metamaterials and related devices has opened a new platform for RVB-based nanofocusing. A single-layer metalens was demonstrated in 2018 to generate and focus RVB simultaneously based on elliptic silicon post arrays, and super-resolution imaging was realized by applying a circular aperture or additional phase distribution to the metalens [73].

For fiber, tip-based nanofocusing has become a hot spot recently for its great meaning for tip-scanning imaging. It is challenging to realize nanofocusing on metalized tips internally illuminated by the conventional LPB for its intrinsic cut-off property at the end of the tip [38, 74]. Complex metallic nanostructures, including semicircular spiral corrugations [75] and spiral conical structures [76] have been proposed to solve

(8) $$\begin{array}{ll} E_{r\text{\_}\text{RVB}}\left(r,\phi ,z\right) & =2A\;\cos\varphi_{0}\overset{\theta_{\text{max}}}{\underset{0}{\int }}P\left(\theta \right)\sin\;\theta \cos\;\theta \\ & \;J_{1}\left(kr\sin\theta \right){\text{e}}^{\text{i}kz\cos\theta }\text{d}\theta \end{array}$$

(9) $$E_{\phi \text{\_}\text{RVB}}\left(r,\phi ,z\right)=0$$

(10) $$\begin{array}{ll} E_{z\text{\_}\text{RVB}}\left(r,\phi ,z\right) & =i2A\;\cos\varphi_{0}\overset{\theta_{\text{max}}}{\underset{0}{\int }}P\left(\theta \right)sin^{2}\;\theta \\ & \;J_{0}\left(kr\sin\theta \right){\text{e}}^{\text{i}kz\cos\theta }\text{d}\theta \end{array}$$

(11) $$E_{r\text{\_}\text{AVB}}\left(r,\phi ,z\right)=0$$

(12) $$\begin{array}{ll} E_{\phi \text{\_}\text{AVB}}\left(r,\phi ,z\right) & =2A\;\sin\;\varphi_{0}\overset{\theta_{\text{max}}}{\underset{0}{\int }}P\left(\theta \right)\sin\;\theta \\ & \;J_{1}\left(kr\sin\theta \right)e^{\text{i}kz\cos\theta }d\theta \end{array}$$

(13) $$E_{\text{z}\text{\_}\text{AVB}}\left(r,\phi ,z\right)=0$$

the problem of LPB, but another problem of poor energy efficiency arises. RVB has been theoretically and experimentally verified [39, 43, 77], [78], [79], [80], [81] to be a point of penetration and breach to overcome the cut-off weakness of LPB with no need for complex nanostructures mentioned above. RVB can be generated in an optical fiber by coupling RVB propagated in free space into a fiber [78] or directly converting LPB guided in fiber into RVB [82]. The latter often uses an index-modulated fiber grating induced by mechanical stress [83], [84], [85] or acoustic signal [86], [87], [88], etc.

The special polarization and focusing characteristics make CVBs have wide and important applications in fields of super-resolution imaging [89], [90], [91], [92], [93], surface-enhanced Raman spectroscopy [94], [95], [96], [97], [98], micro-nanofabrication [99], [100], [101], optical tweezers [102], [103], [104], etc. In this paper, we focus on CVB-based super-resolution imaging.

## 3 Strategies for CVB-assisted super-resolution imaging

**Figure 3:**
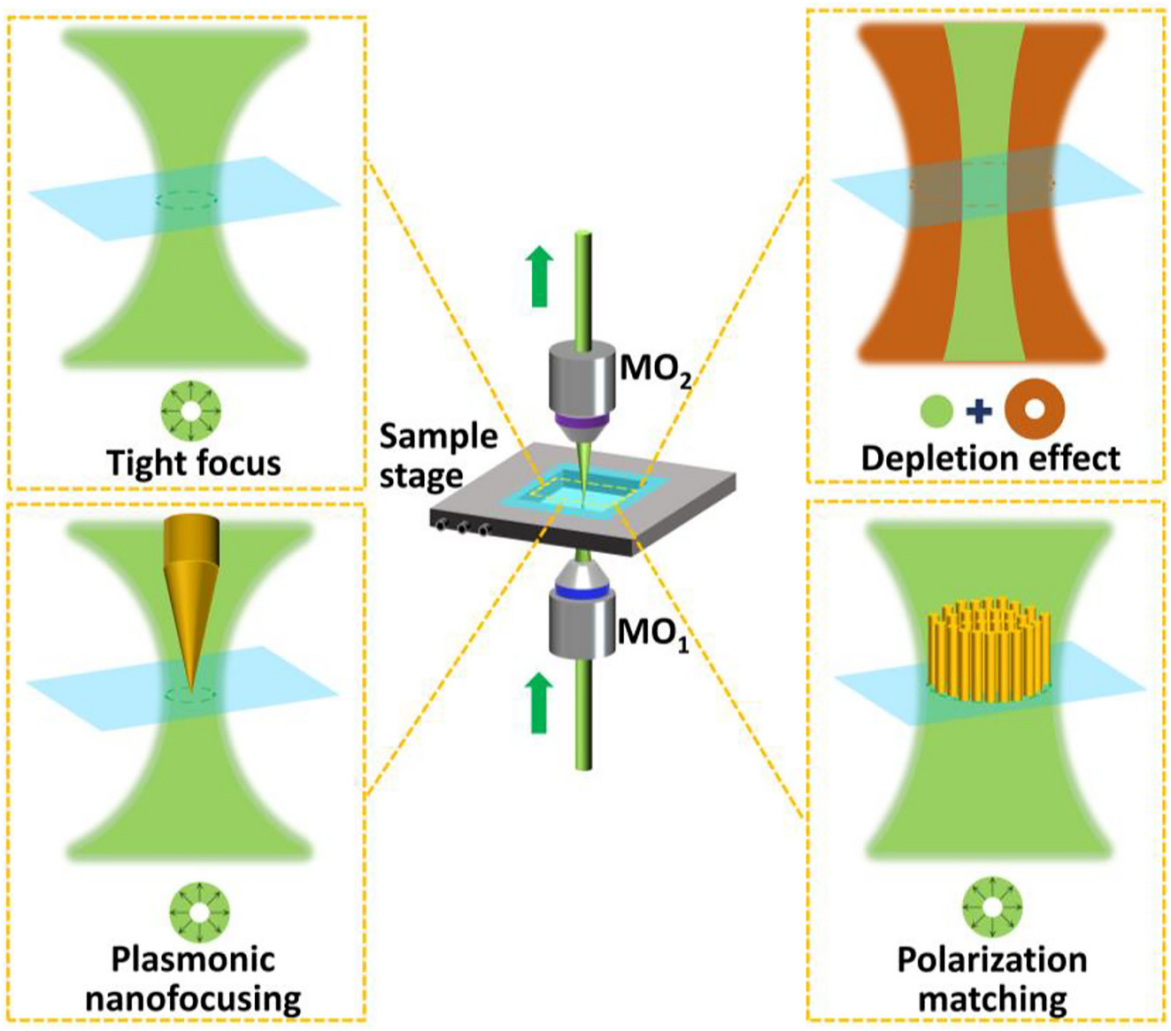
Strategies for CVB-assisted super-resolution imaging.

In near-field optical microscopy, CVBs assist resolution enhancement via tight focusing, excitation enhancement, and polarization matching. The first strategy for CVB-assisted super-resolution is to create a sub-diffraction excitation volume through tight focusing [36, 64, 105, 106] and plasmonic nanofocusing [107], as shown in Figure 3. The second strategy for CVB-assisted super-resolution is excitation enhancement based on tapered plasmonic waveguides [108, 109], which can be realized by polarization matching [110], because the excitation of localized surface plasmon modes [111, 112].

Here, plasmonic nanofocusing can be achieved by two classes of methods. One is employing a laser beam to focus directly on a tapered plasmonic waveguides in free-space to excite localized surface plasmons at the taper apex [108, 109]. The other is utilizing an optical waveguide mode light to internally illuminate aperture-less metalized tips to excite localized surface plasmons [41, 43]. The former has a shortcoming of inevitable background noise for the mismatch between the nanoscale tip apex and the microscale focal spot. The latter can overcome the background problem, but the conventional linearly polarized waveguide mode cannot excite surface plasmon polaritons concentrating at the tip apex. RVBs have been verified to realize plasmonic nanofocusing on the metalized fiber tip and benefit resolution improvement of tip-scanning near-field microscopy.

Polarization matching is very important for plasmonic tip-based near-field microscopy, because it is the prerequisite condition to efficiently excite surface plasmon polaritons. Only when the polarization direction is perpendicular to the metal-dielectric interface, surface plasmon polaritons can be generated. Considering metal nanostructures like nanotips, the strong longitudinal field component of illumination focusing field along the tip axis has the best-matched polarization direction, resulting in efficient excitation of localized surface plasmon modes with nanoscale “hot spot” [113] and considerable electric-field enhancement. RVBs that have strong longitudinal field component under tight focusing is identified to possess the best-matched polarization direction for nanotip-based near-field microscopy. Besides plasmonic tip-based near-field microscopy, nonlinear imaging techniques also benefit from polarization matching for dependency of nonlinear response to the polarization direction.

In far-field optical microscopy, which refers to STED microscopy, subtraction microscopy, and superoscillation microscopy in this paper, tight focusing of CVBs to generate desired patterns (e.g. doughnut or subdiffraction-limited spot) is the key strategy to achieve super-resolution. First, in STED, the basic principle is depletion effect. The excitation beam is overlaid by a doughnut-shaped depletion beam with an intensity profile that is ideally zero in the central subregion of the Gaussian excitation spot and increases toward the periphery. De-excitation of fluorophores by stimulated emission across the entire observation volume except in the very center spatially confines the excitation spot [114], [115], [116], [117]. Therefore, the focal field of excitation beam and depletion beam, and their alignment largely dominate the performance of STED microscopy. RVBs and AVBs have been respectively applied as pump beam and depletion beam by manipulating the focal field, which can realize both lateral and axial resolution enhancement and orientation microscopy [118], [119], [120].

Second, CVB-assisted tight focusing in subtraction microscopy provides super-resolution. Subtraction microscopy is a typical far-field microscopy based on subtraction of two images which are respectively acquired with closed and open pinhole [121, 122], or with different point spread functions (PSFs) [123], etc. Tight focusing (NA = 0.90) of CVBs has been applied in subtraction imaging to suppress the negative side lobe for resolution enhancement [121].

Third, CVB allows for generation of sub-diffraction patterns in superoscillation microscopy. Superoscillation microscopy [124] is a newly developed super-resolution far-field technology. The key to superoscillation imaging is to meet superoscillation criterion that a band-limited function locally oscillating faster than its fastest Fourier component and generate a nanoscale “hot spot.”Superoscillation microscopy has also employed tightly focused RVBs with higher-order transverse mode for the resolution issue. Based on vector diffraction theory, RVBs under tight focusing has been verified to help to meet superoscillation criterion and contribute to reducing illumination volume for super-resolved imaging. Besides, tight focusing also benefits STED microscopy, tip-scanning imaging and nanotip-based nonlinear imaging, as mentioned before.

The aforementioned strategies do not always function independently. Some hybrid-mode imaging techniques have been exploited to obtain the advantages of all strategies. Therefore, tight focusing, depletion effect, plasmonic nanofocusing, and polarization matching are usually overlapping in super-resolution cases. In other words, excitation-volume decrease and excitation enhancement often occur simultaneously and do not always function independently.

## 4 Roadmap of CVB-assisted super-resolution imaging techniques in the past two decades

**Figure 4:**
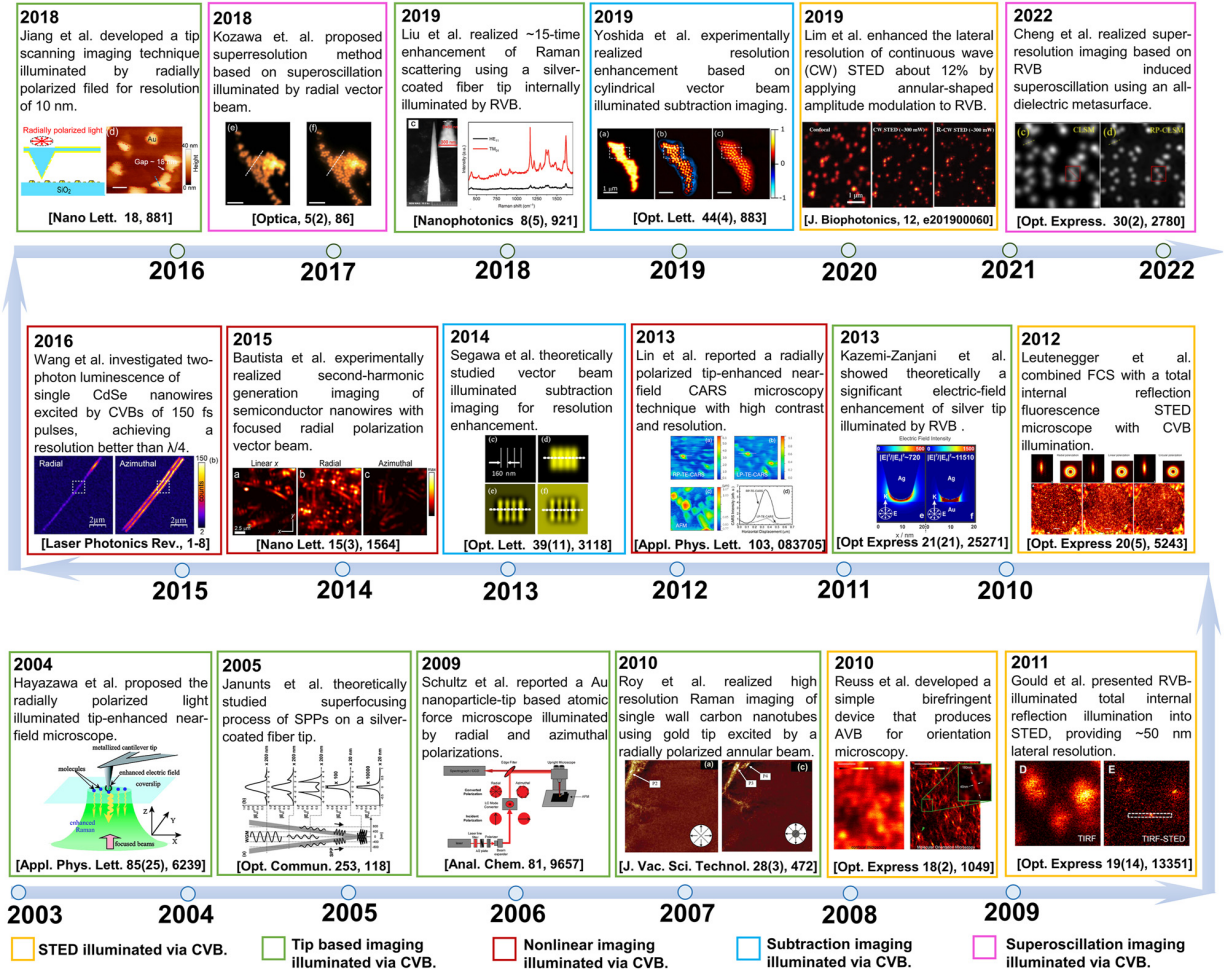
Roadmap of the 20-year development of CVB-based super-resolution imaging from 2003 to 2022. Adapted from Ref. [37]. Copyright 2004 American Institute of Physics. Adapted from Ref. [38]. Copyright 2005 Elsevier B.V. All rights reserved. Adapted from Ref. [125]. Copyright 2009 Am. Chem. Soc. Adapted from Ref. [126]. Copyright 2010 American Vacuum Society. Adapted from Ref. [137]. Copyright 2010 Optical Society of America. Adapted from Ref. [45]. Copyright 2011 Optical Society of America. Adapted from Ref. [46]. Copyright 2012 Optical Society of America. Adapted from Ref. [42]. Copyright 2013 Optical Society of America. Adapted from Ref. [153]. Copyright 2013 AIP Publishing LLC. Adapted from Ref. [149]. Copyright 2014 Optical Society of America. Adapted from Ref. [154]. Copyright 2015 American Chemical Society. Adapted from Ref. [156]. Copyright 2016 by WILEY-VCH Verlag GmbH & Co. KGaA, Weinheim. Adapted from Ref. [44]. Copyright 2017 American Chemical Society. Adapted from Ref. [91]. Copyright 2018 Optical Society of America under the terms of the OSA Open Access Publishing Agreement. Adapted from Ref. [43]. Copyright 2019 Wending Zhang, Ting Mei et al., published by De Gruyter. Adapted from Ref. [92]. Copyright 2019 Optical Society of America. Adapted from Ref. [118]. Copyright 2019 WILEY-VCH Verlag GmbH & Co. KGaA, Weinheim. Adapted from Ref. [139]. Copyright 2022 Optica Publishing Group under the terms of the Optica Open Access Publishing Agreement.

In the past two decades, CVB has been applied in super-resolution optical microscopy based on the above-mentioned strategies. Figure 4 shows a roadmap of the 20 year development of CVB-based super-resolution imaging from 2003 to 2022, including both near-field and far-field techniques.

### 4.1 Near-field microscopy

RVB plays an important role in tip-scanning microscopy, including both external and internal illumination for metal tip and fiber-based tip, respectively. In free space, Dorn et al. first experimentally verified in 2003 that RVB can realize sub-diffracted focusing [36]. In the next year, Hayazawa et al. applied RVB to tip-enhanced Raman spectroscopy through tightly focusing RVB onto a silver-coated cantilever tip via a high numerical aperture objective lens, leading to higher sensitivity for detecting spectroscopy of adenine nanocrystals [37]. In 2008, Stanciu et al. developed a parabolic-mirror-based TERS system and obtained distinguishable images of a 60-nm Au sphere by RVB illumination compared to that by LPB and AVB [72]. Th Schultz et al. reported in 2009 that an Au nanoparticle-tip-based atomic force microscope illuminated by radial and azimuthal polarizations [125]. In 2010, Roy et al. realized high resolution Raman imaging of single-wall carbon nanotubes using gold tip excited by a radially polarized annular beam [126].

For fiber-based near-field microscopy, it was theoretically proposed that a radially polarized waveguide mode can converge towards the very end of a noble metal-coated fiber tip and create a strong field enhancement localized at the apex [38, 127]. The conversion mechanism of RVB waveguide modes into surface plasmon polaritons (SPPs) in a metallic fiber tip has been theoretically studied in 2007 [74] and in 2012 [39]. With the development of RVB generation technologies, RVB start to be used in experiment for nanoscale near-field source [43, 78], [79], [80], [81]. Tugchin et al. experimentally studied the excitation process of the radially polarized conical surface plasmon polariton and the reverse process [79]. As shown in Figure 4, it was in 2018 that fiber-tip-based near-field microscopy practically realized super-resolution imaging with CVB-based illumination. Jiang et al. utilized a gold-coated SiO_2_ tip with six annular slits on the coating as scanning tip, and RVB as illumination source, to obtain a resolution of 10 nm [44]. In 2019, Kim et al. integrated a tapered optical fiber with a sharp-tip Ag nanowire to form a new-typed plasmonic tip, which successfully converted the optical LPB mode to RVB plasmon mode and resulted in plasmonic nanofocusing and further applied in tip-scanning imaging [92]. This method is reported to have spatial resolution of 1 nm.

### 4.2 Far-field microscopy

For far-field super-resolution microscopy, early in 1994, doughnut-shaped beam was applied in STED and achieved diffraction-limited resolution [128], [129], [130], [131], [132], [133], [134], for which Betzig, Hell and William were jointly awarded the Nobel Prize in Chemistry 2014. In STED microscopy, the commonly used depletion beam is circularly-polarized vortex beam [129, 135]. It was reported in 2007 that RVB can generate a three-dimensional (3D) dark focal spot [47] for STED applications. As exhibited in Figure 4, in 2010, Reuss et al. utilized AVB as depletion beam, which realized orientation imaging through offering orientation information of fluorescent molecules with advantages of convenient beam-alignment [136]. In 2011, it was verified by Gould et al. that RVB could offer a way for super-resolution by being applied as pump beam to total internal reflection fluorescence in STED microscopy [45]. In the same year, 3D super-resolution spot was achieved by overlapping two beams as STED beam [137]. In 2012, AVB was proposed to generate a sharper fluorescent super-resolution spot, and verified to have the most efficient STED action compared to RVB, LPB, and circularly polarized vortex beam [46, 119]. In 2019, RVB was used in continuous wave (CW) STED microscopy to enhance the lateral resolution by ∼12% with annular-shaped amplitude modulation [118].

Besides STED microscopy, subtraction microscopy and superoscillation imaging are also nonnegligible far-field techniques that have adopted CVB as illumination source to seek for resolution enhancement. In 2014, Segawa et al. theoretically studied vector beam illuminated subtraction imaging for resolution enhancement [138], and lately in 2019, Yoshida et al. experimentally achieved a spatial resolution of ∼100 nm based on CVB-illuminated subtraction imaging without the degradation of image quality [91]. The report about application of CVBs to super-resolution imaging is in 2018 that Kozawa et al. proposed super-resolution superoscillation microscopy using RVBs as illumination, through which lateral resolution close to 100 nm could be obtained for practical confocal laser scanning microscopy with visible light [90]. The recent boom of metasurface and metamaterials brings about new advancement of superoscillation. Cheng et al. reported in 2022 that through an all-dielectric metasurface, RVB could generate and induce superoscillation, leading to super-resolution imaging with 2-fold lateral resolution enhancement [139].

Moreover, nonlinear imaging has also adopted CVB as illumination source for nonlinear effects and resolution enhancement [140]. Here, nonlinear effects, which generally include two-photon-excited luminescence and fluorescence [141,142], coherent anti-Stokes Raman scattering (CARS) [143] second-harmonic generation (SHG) [144], third-harmonic generation (THG) [145], and often occur under strong laser illumination with high power density. Yew et al. studied the SHG characteristics under illumination of tightly focused RVB in 2007, which found radial polarization distribution of the output SHG after collimation and its independence on analyzer angle [146]. A few years later, researches on THG and SHG of silver and gold nanocones using tightly focused CVBs were carried out [147], [148], [149]. Besides, nonlinear effects of silicon or plasmonic oligomers excited by CVBs have also attracted much attention recently [150], [151], [152], but they concentrate on the rule of the interaction between CVB and the designed structures rather than nonlinear imaging. It was in 2013 that Lin et al. reported a radially polarized tip-enhanced near-field CARS microscopy technique with high contrast and resolution [153], which firstly realized nonlinear imaging using RVB illumination, as shown in Figure 4. Two years later, Bautista et al. experimentally realized SHG imaging of semiconductor nanowires with focused RVB [154], based on which three-dimensional imaging technique were further developed and realized SHG imaging of 3D nanowires by the same group [155]. Besides, two-photon luminescence and SHG of single CdSe nanowires excited by tightly focused cylindrical vector beams of 150 fs pulses was addressed by Wang et al. in 2016 and 2017, respectively, where an optical resolution could be better than *λ*/4 [156, 157].

During the past two decades, the applications of CVB in field of super-resolution optical microscopy has covered tip-scanning imaging, CARS imaging, SHG imaging, two-photon luminescence microscopy, STED microscopy, subtraction imaging, superoscillation imaging, etc. Next, typical CVB-assisted super-resolution imaging techniques will be introduced, including describing the theoretical methods, the experimental set-up, advantages and disadvantages, etc.

## 5 Typical CVB-assisted super-resolution imaging techniques

### 5.1 Near-field microscopy

#### 5.1.1 Tip scanning imaging

Tip-based scanning microscopy provides nondestructive techniques for detecting structural and morphology information of molecules with spatial resolution beyond the optical diffraction limit. The key component of tip scanning imaging system is a tip with a sharp apex in the nanoscale, which can generate LSPR based on the “lightning rod” effect [158], [159], [160]. In other words, nanometer-sized hotspots [161], [162], [163] can be generated and contribute to the enhancement of electric field and spatial resolution. Up to now, many attempts have been made to improve the spatial resolution of TERS. Different shapes include metal coated fiber tips [164], nanograting modified metal tips [165], gold-filled hybrid tips [78, 80], pyramid-shaped tips [166], symmetry-broken spiral tapers [76], campanile near-field probes [167] etc. have been developed to realize nanofocusing in order to be further applied in super-resolution imaging. Correspondingly, different nanofabrication technologies, e.g., electrochemical etching [28,168,169], photochemical deposition [170], [171], [172], vapor coating [173] etc., have been exploited and optimized for precise control of tip shapes. More importantly, the new-typed illumination modes offer a new avenue for resolution promotion due to the dependence of LSPR excitation on the polarization of illumination light fields [174]. Thus, CVBs have been adopted as illumination source for tip-scanning microscopy including AFM-based [44] and hybrid fiber tip-based [92] near-field super-resolved imaging.

**Figure 5:**
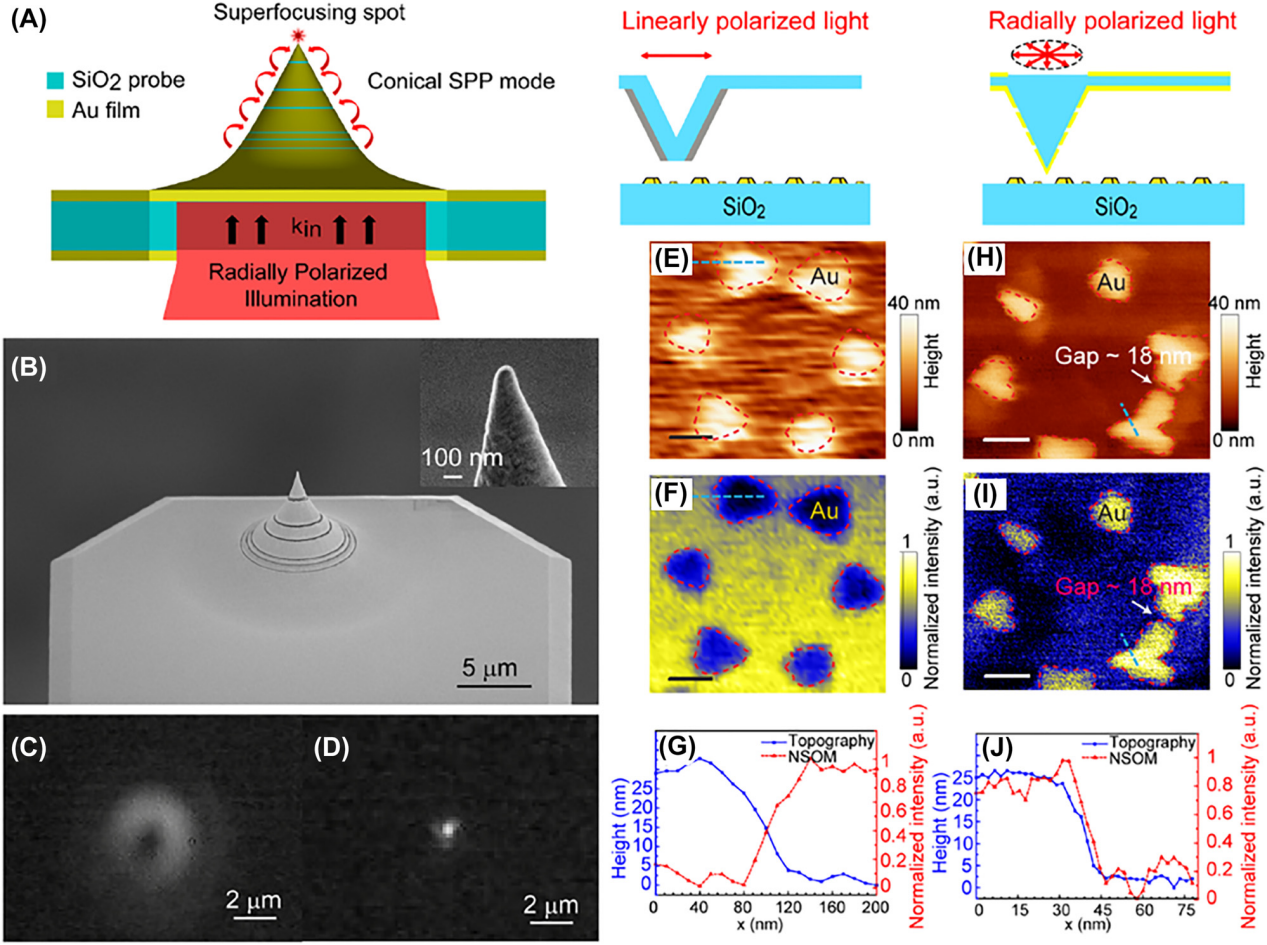
Scanning near field imaging illuminated via RVB. (A) Principle of the super-focusing mode excitation at the tip apex under RVB illumination. (B) Scanning electron microscope (SEM) images of the Au-coated SiO_2_ AFM probe with six annular slits in the Au film. (C) Image of the focused RVB excitation recorded via a high numerical aperture objective lens (100×, NA = 0.8). (D) Image of superfocusing of SPP at the tip apex recorded via a high numerical aperture objective lens (100×, NA = 0.8). (E) Topography and (F) the corresponding near-field scanning optical microscopy image of Fischer’s pattern via a commercial tip in the case of linear polarized light illumination. (G) Height profile (blue) and corresponding NSOM intensity profile (red) of the sharpest edge obtained via a commercial tip. (H) Topography and (I) the corresponding near-field scanning optical microscopy image of Fischer’s pattern via the fabricated plasmonic tip in the case of RVB illumination. (J) Height profile (blue) and corresponding NSOM intensity profile (red) of the sharpest edge obtained via the fabricated plasmonic tip. Adapted from Ref. [44]. Copyright 2017 American Chemical Society.

Jiang et al. developed a plasmonic tip with subwavelength annuli to efficiently couple internal radial illumination to SPPs, and thus realize nanofocusing at the apex and topographic resolution of 10 nm with high signal-to-noise ratio [44]. Principle of the super-focusing mode excitation at the tip apex under RVB illumination is shown in Figure 5A. They fabricated a phase-matching plasmonic tip by coating a 120 nm-thick Au film onto the surface of a commercial SiO_2_ AFM probe via sputtering, and then inscribing six annular slits in the Au film via focused ion beam (FIB) milling, as shown in Figure 5B. Through optimizing the locations of the slits, difference between phase delays of the SPP wave propagation and the excitation light propagation in SiO_2_ can be adjusted to be 2*nπ*, and thus Fabry–Perot resonance condition is satisfied, which results in SPP interference and super-focusing at the tip apex. An RVB with a spot size of 6 μm (in Figure 5C) was experimentally utilized to illuminate the tip from the backside, which generates a focus with a size of 400 nm at the focal plane above the tip apex (in Figure 5D).

The configuration was integrated into a near-field scanning optical microscopy system and used to image a standard Fischer’s pattern (60–100 nm sized Au projection pattern of a 600 nm polymethyl methacrylate (PMMA) sphere on glass, where the gap size ranges from 10 to 30 nm as a reference). Figure 5E shows the imaging results obtained by a commercial tip with a ∼80 nm-sized aperture, where the resolution of the topographic image in Figure 5E is 60 ± 10 nm, and that of the darker optical signal in Figure 5F is 48 ± 10 nm. In the case of RVB illumination, the topographic (in Figure 5H) and optical (in Figure 5I) resolutions were both 10 nm, respectively, which is improved a lot compared to that of the commercial tip illuminated via LPB. Moreover, it was found that Figure 5G and J have a reverse tone which is likely because the commercial aperture tip supports the transverse dipole, while the plasmonic tip illuminated via RVB supports the longitudinal dipole at the apex. In addition, the proposed configuration was applied to characterize the propagation and interference of the plasmonic lens and the signal-to-noise ratio is as high as ∼18.2.

This work [44] realizes high-resolution imaging by a gold-coated SiO2 tip illuminated via RVB, which experimentally verifies the nanofocusing and resolution-improving capability for RVB illuminated near-field imaging. However, a gold-coated SiO_2_ tip with nanoscale annular slits is needed, making it difficult and expensive to prepare.

**Figure 6:**
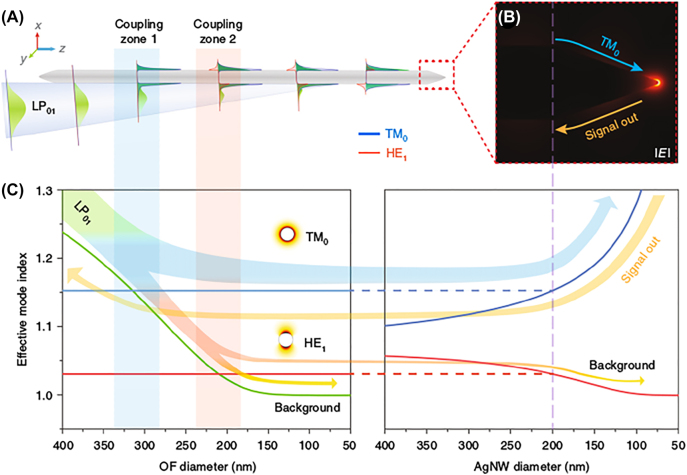
Principle of linear fiber mode converting into SPP TM_0_ mode by fiber taper integrated with a sharp Ag nanowire. (A) The phase-matching zones for linear fiber mode converting into SPP TM_0_ mode. (B) Simulation of the nanofocusing at apex of the Ag nanowire (angle 37°) under TM_0_ illumination from the left. The light wavelength kept 532 nm for all calculations. (C) Calculated effective mode index of linear fiber mode (LP_01_, green), TM_0_ mode (blue line) of a Ag nanowire and HE_1_ mode (red line) of a Ag nanowire varied with fiber diameters (Left). Effective mode indices of the TM_0_ mode (blue) and HE_1_ mode (red) varied with Ag nanowire diameters (Right). Adapted from Ref. [92]. Copyright Kim S, Yu N, Ma XZ, Zhu Y, Liu Q, Liu M, Yan R, under exclusive licence to Springer Nature Limited 2019.

In addition, hybrid fiber tip-scanning microscopy illuminated with RVB was proposed to enable resolution enhancement [92]. As shown in Figure 6A, the hybrid tip consisting of fiber taper and silver nanowire was verified to have interesting physics that an input LPB mode in the optical fiber can be converted to radial SPP mode (TM_0_) in the silver nanowire, which can obtain nanofocusing by two consecutive steps [92]. It can be seen clearly from Figure 6A–C that mode-coupling process occurred between the fiber taper and the silver nanowire so that TM_0_ mode of the nanowire can be selectively excited with the linear fiber mode (LP_01_) from the fiber taper. And then the quasi-adiabatic nanofocusing can be realized through further compressing the TM_0_ mode via the sharp apex of the silver nanowire.

**Figure 7:**
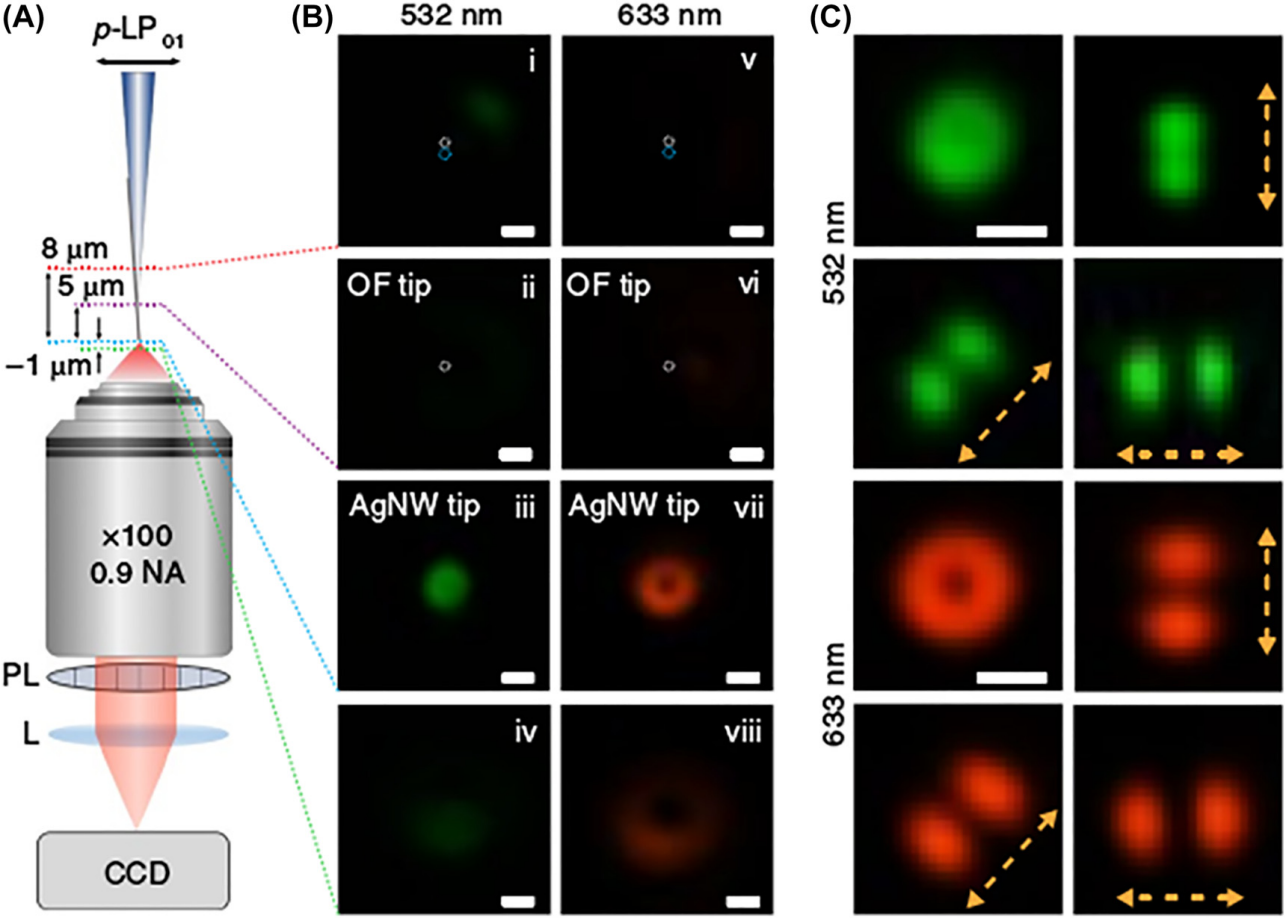
Experimental verification of TM_0_ mode excitation and nanofocusing. (A) Sketch map of high-magnification optical microscope for characterizing converter process of linear fiber mode (LP_01_) to radial SPP mode (TM_0_). PL: polarization analyzer; L: lens; CCD: charge coupled device. (B) Images of different focal plane located along the hybrid fiber tip: near the coupling zones (i, v); at apex of the optical fiber taper (ii, vi); at apex of the Ag nanowire tip (iii, vii); defocused by 1 μm (iv, viii). Excitation wavelengths are 532 and 633 nm respectively. (C) Polarization examination results of B (iii) and (vii). Yellow dashed arrows illustrate the transmission axis of PL. Scale bars, 500 nm. Adapted from Ref. [92]. Copyright Kim S, Yu N, Ma XZ, Zhu Y, Liu Q, Liu M, Yan R, under exclusive licence to Springer Nature Limited 2019.

The experimental equipment shown in Figure 7A was used to verify the nanofocusing process with the proposed hybrid tip. Figure 7B exhibits vertical sectioning images corresponding to different positions of the micro-objective (NA = 0.9) focal plane marked in Figure 7A with a dashed line. Figure 7B(i, ii) and (v, vi) exhibits that there is almost no light scattered near the coupling zones and from the optical tip, revealing that nearly all of the energy has been coupled into the nanowire. Figure 7B(iii, vii) are the imaging results when the focal plane of the micro-objective marked in Figure 7A is located at the silver nanowire tip, which demonstrates that TM_01_ mode has been excited at the nanowire tip for the doughnut-like shape of the radially polarized far-field scattering, and for the corresponding polarization characteristics measured in Figure 7C. This hybrid configuration ingeniously realized plasmonic nanofocusing, and was further applied in TERS mapping by converting a basic portable STM to a lens-free TERS system, where the optical fiber was coated with 100-nm-thick film to make it conductive and compatible with STM. The spatial resolution of the developed TERS system based on the TM_0_-mode nanofocusing via the hybrid tip was verified to be as high as 1 nm [92]. Moreover, the developed technique has broad working bandwidth and high external nanofocusing efficiency of ∼50%.

This work [92] develops a new approach of RVB-illuminated tip-scanning microscopy by mode converter to ingeniously obtain nanofocusing with no need for complex experimental setups for RVB generation. However, it is difficult to possess high stability and repeatability for the special configuration of the hybrid tip.

#### 5.1.2 Nonlinear imaging

Nonlinear imaging is a powerful microscopic technique based on optical nonlinear effects like CARS [143], SHG [144], THG [145], etc. To date, a large number of researches have been done to improve the resolution. Considering that different nanostructured nonlinear materials have different nonlinear response to the incident light with polarization directions, which depends on the match degree between structure orientations and polarization directions, CVBs have been applied in nonlinear microscopy to expand new capabilities. CVBs with radial and azimuthal polarization distributions have different interactions with different samples, and the nonlinear imaging technique can be benefitted a lot from the optimization of CVB illumination methods.

CARS microscopy utilizes four-wave mixing process to realize Raman imaging based on third-order nonlinear effects. Similar to TERS, CARS has advantages of label-free detection, chemical specificity, molecular identification, etc., which has become a powerful tool in biological and biomedical characterizing [175, 176]. The typical CARS system has a collinear geometry of the pump and Stokes beams with tight focusing under microscope. The resolution largely depends on the overlap of the pump and Stokes beams, and generally limited by optical diffraction limit. To date, many efforts have been done to improve the resolution via enhance the nonlinear signal and reduce the efficient illumination region [177], [178], [179].

**Figure 8:**
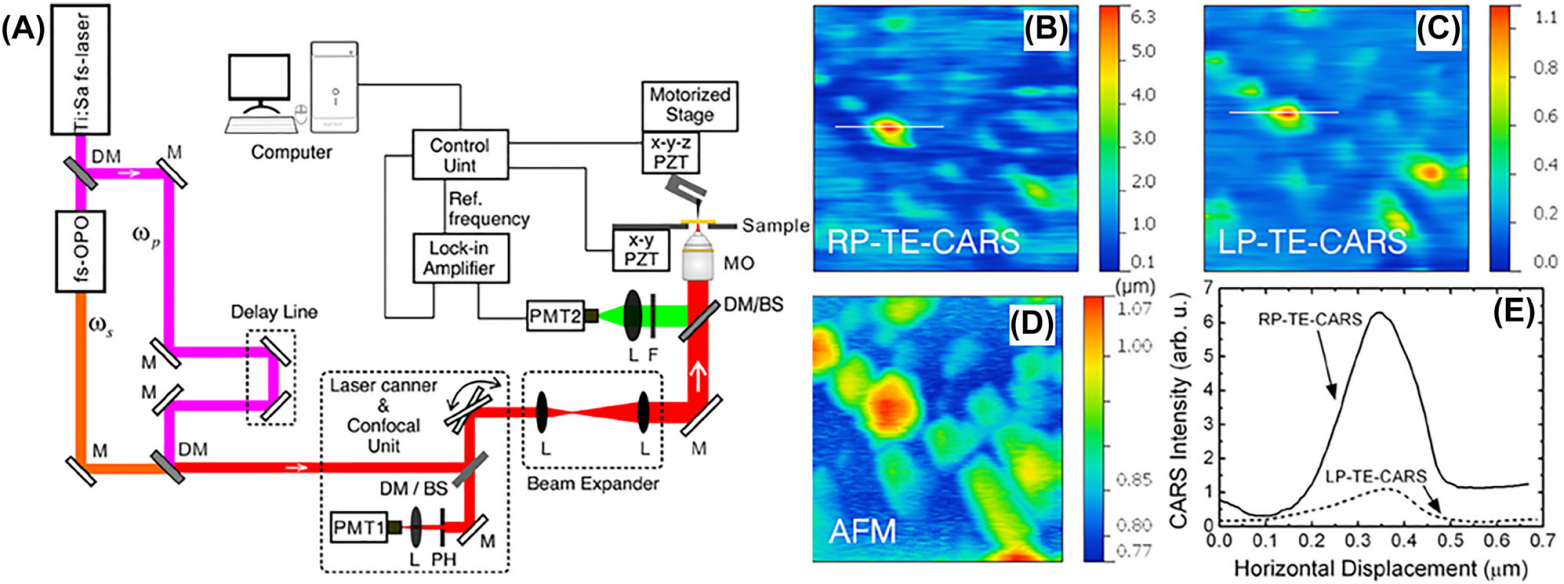
Coherent anti-Stokes Raman scattering microscopy illuminated via CVB. (A) Schematic of the radially polarized tip-enhanced near-field CARS microscope. DM, dichroic mirror; L, lens; M, mirror; RP, radial polarizer; MO, microscope objective; F, filter; PZT, piezoelectric transducer; PH, pinhole; BS, beam splitter; PMT, photomultiplier tube. (B) Radially polarized near-field TE-CARS images of polystyrene beads with sizes varying from 100 to 300 nm. The image size is 1.5 × 1.5 μm^2^. (C) Linearly polarized near-field TE-CARS images of the same samples with (A). The image size is 1.5 × 1.5 μm^2^. (D) AFM images of the same samples with (A). The image size is 1.5 × 1.5 μm^2^. (E) Intensity profiles along the white lines in (B) and (C). Adapted from Ref. [153]. Copyright 2013 AIP Publishing LLC.

Huang’s group applies the radially polarized pump and Stokes laser beams to CARS microscopy technique for high-contrast vibrational imaging through combining CARS with the tip-enhanced microscopy [153]. CARS signal of samples was excited by the tightly-focused pump and Stokes laser beams and further enhanced by a metallic probe near the sample surface due to the significant electric-field enhancement. The optical system of CARS microscopy illuminated via CVB is shown in Figure 8A, where the back-scattered signal is measured by the gold-coated tip controlled by a homemade tuning-fork-based atomic-force-microscope (AFM) scanning unit. A femtosecond Ti: Sapphire laser was used as a laser source to generate pump beam and Stokes beam by dividing into two parts via a beam splitter. Note that an optical parametric oscillator (OPO) was needed to convert the output femtosecond laser into the Stokes beam for CARS microscopy. The liquid-crystal-based radial polarization converters [180] were used to generate the radially polarized pump and Stokes light fields. The generated radially polarized pump and Stokes light beams are collinear and focused onto the sample through a high NA microscope objective, and simultaneously delivered into a confocal laser scanning microscope to realize image recording. The locking amplifier was applied to amplify and collect the reflected tip enhanced-CARS or NSOM signal. Figure 8B and C shows the CARS images of polystyrene beads with a size of 100–300 nm illuminated by radially polarized and linearly polarized beams, respectively. Figure 8D and E exhibit the corresponding AFM image and intensity profiles along the lines in Figure 8B and C, respectively. It can be seen that the CARS signal illuminated by RVB is 6-fold higher than that using linearly polarized laser excitation, and the nanoscale polystyrene beads are more distinguished under RVB illumination compared with LPB illumination. In addition, mitochondria were characterized by AFM and the RVB-illuminated CARS, where an obvious hole with a size of 80 nm can be seen in the mitochondria by RVB-illuminated CARS but cannot be distinguished by AFM. The signal-to-background ratio can be improved by ∼3 times compared to LPB. It reveals that the tightly focused radially polarized light can be used to efficiently enhance the electric field near the metallic tip and further to significantly improve the CARS signal via near-field enhancement, which offers a brand-new way for super-resolution CARS imaging.

This work [153] strengthens CARS signal and improves the imaging resolution by combing the tip-enhanced nonlinear effects illuminated by RVB with conventional CARS microscopy, which opens a new platform for super-resolution imaging. Although the resolution has been enhanced, the complexity of the optical system has also increased. Moreover, it is difficult to realize the alignment of tip axis, pump beam, and Stokes beam.

**Figure 9:**
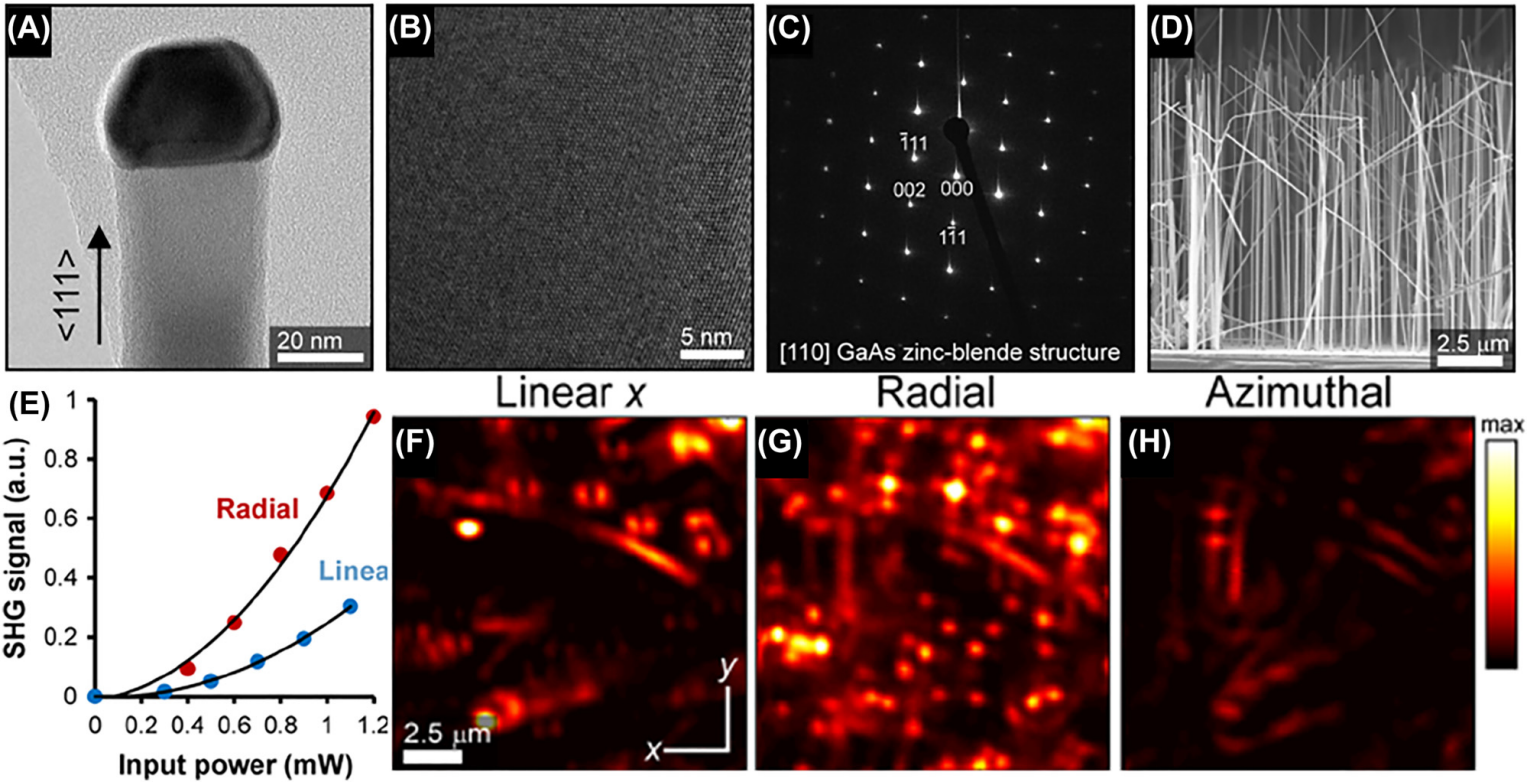
Second-harmonic generation images of GaAs nanowires excited by CVB. (A, B) TEM characterizing results of a GaAs nanowire grown on a GaAs substrate. (C) Typical indexed ED pattern taken at different sections along the nanowire axis. (D) SEM image of the GaAs nanowires used in the SHG imaging (side view). (E) Power dependence of the SHG signal from an individual GaAs nanowire using focused RP and LP. (F) Far-field SHG images of vertically aligned GaAs nanowires (diameter = 40 nm, length = 10 μm) illuminated by LP along *x* direction. (G) Far-field SHG images illuminated by RP under the same experiment settings with (F). (H) Far-field SHG images illuminated by AP under the same experiment settings with (F). Adapted from Ref. [154]. Copyright 2015 American Chemical Society.

Besides CARS microscopy, SHG imaging is also a powerful nonlinear imaging technique and morphology characterization tool for its inherent sensitivity to structural symmetry and label-free characteristics [181]. SHG is the most-common optical nonlinear process that is generated under high-power excitation. However, high excitation power like femtosecond laser irradiation may bring about high-order absorption. Therefore, it is necessary to explore the method of efficient excitation with low-power laser irradiation. Bautista et al. applied the three-dimensional vector fields to SHG imaging to seek new capabilities due to the fact that the image contrast often varies with the nonlinear susceptibility tensors. GaAs nanowires have been investigated by SHG imaging illuminated with focused vector beams, the results of which are shown in Figure 9 [154]. A custom-built point-scanning SHG microscope operating in reflection was used in the study, where a mode-locked femtosecond Nd:glass laser (wavelength 1060 nm, pulse length 200 fs, repetition rate 82 MHz) was utilized as the excitation source, an infinity-corrected (50×, 0.8 NA) and strain-free micro-objective was used to focus the beam onto the sample and collect the reflected SHG signals, a dichroic mirror was used to discriminate the unwanted reflected signals, a three-axis piezo-scanning stage was used to adjust the sample position. The RVB or AVB were generated before the objective by a polarization mode converter (ARCoptix, S.A.) with built-in phase compensation in tandem with a spatial Fourier filter. In addition, the appropriate spectral band filters, a tube lens, and a cooled photomultiplier tube were used to improve the signal-to-noise ratio.

Figure 9A and B shows the morphology of a GaAs nanowire grown on a GaAs substrate characterized by transmission electron microscope (TEM) [154], where the growth direction of the nanowire is indicated by the arrow in Figure 9A. Figure 9C exhibits the typical indexed electron diffraction (ED) pattern taken at different sections along the nanowire axis, revealing a pure zinc-blende structure. As depicted in Figure 9D, scanning electron microscope (SEM) image of the GaAs nanowires from the side view demonstrates the vertical arrangement of nanowires. Figure 9E shows the power dependence of the SHG signal from an individual GaAs nanowire illuminated by RVB and LPB, where both curves represent quadratic dependence. The SHG intensity excited by RVB is enhanced of 3–5-folds and 30-folds, respectively, in experiment and simulations compared to LPB. Figure 9F–H exhibit the far-field SHG images of vertically aligned GaAs nanowires with a diameter of 40 nm and length of 10 μm illuminated by LPB along *x*-direction, RVB, and AVB respectively, where beams were focused onto the tip of the nanowires, and other experiment settings were kept constant. Note that the average power in the imaging experiments is less than 2 mW. It can be seen from Figure 9F that in the case of LPB illumination, resemble two-lobed patterns with a dark spot at the center are obtained, where the two-lobed patterns are symmetric with respect to the incident LPB axis, which is due to the distribution of the longitudinal component of a focused LPB beam [182]. In the case of RVB illumination, as depicted in Figure 9G, the point-like distribution at the location of the nanowires is obtained, and the generated SHG signals are stronger than that under LPB, which is due to the strong longitudinal field at the center of the focused RVB. In the case of AVB illumination, as depicted in Figure 9H, no significant SHG patterns from the vertical nanowires is obtained due to the absence of any longitudinal fields at the focus. This work demonstrates the underlying physics that SHG from oriented nanowires is mainly driven by the longitudinal field along the nanowire growth axis, revealing the superiority of RVB to characterize such nanowires compared to LPB, which could be further applied in the growth monitoring, morphology characterizing, etc.

This work [154] proposed a novel nonlinear-imaging method for vertically oriented GaAs nanowires with 40 nm diameter by RVB illumination. However, it is not a general super-resolution imaging method for all nonlinear structures and orientations, which may limit its applications.

### 5.2 Far-field optical microscopy

#### 5.2.1 STED microscopy

STED microscopy realizes super-resolution imaging by taking the dynamic transition of fluorophores into consideration during the image-formation process [183]. Through simultaneous illumination of two coaxial laser beams called excitation laser and STED laser, where the STED beam has a doughnut-like cross-section, and the STED beam has a longer wavelength than that of the excitation laser beam, fluorophores can be excited from the ground state to an excited-state by excitation laser, and de-excited via stimulated emission by STED laser [16, 184], [185], [186].

STED has been applied in fields of chemistry [187], biology [188], materials [189], etc. for its advantages of super-resolution, high sensitivity, optical sectioning, etc. However, it is difficult to simultaneously acquire high lateral and axial resolution. To solve the problem, RVB has been applied as excitation beam in total internal reflection fluorescence (TIRF) STED and achieved a lateral resolution of ∼50 nm and an axial confinement of ∼70 nm simultaneously [45, 46]. In addition, AVB has been reported to be used as depletion beam through a simple birefringent device with advantages of convenient beam alignment [136, 190], which can realize orientation imaging by offering orientation information of fluorescent molecules due to the tangential polarization distribution and special focusing characteristics. Moreover, considering the tightly-focusing properties of RVB that an ultra-small focusing spot and a large longitudinal field component can be generated, RVB are promising to act as an effective excitation beam of STED for both lateral and axial resolution enhancement.

**Figure 10:**
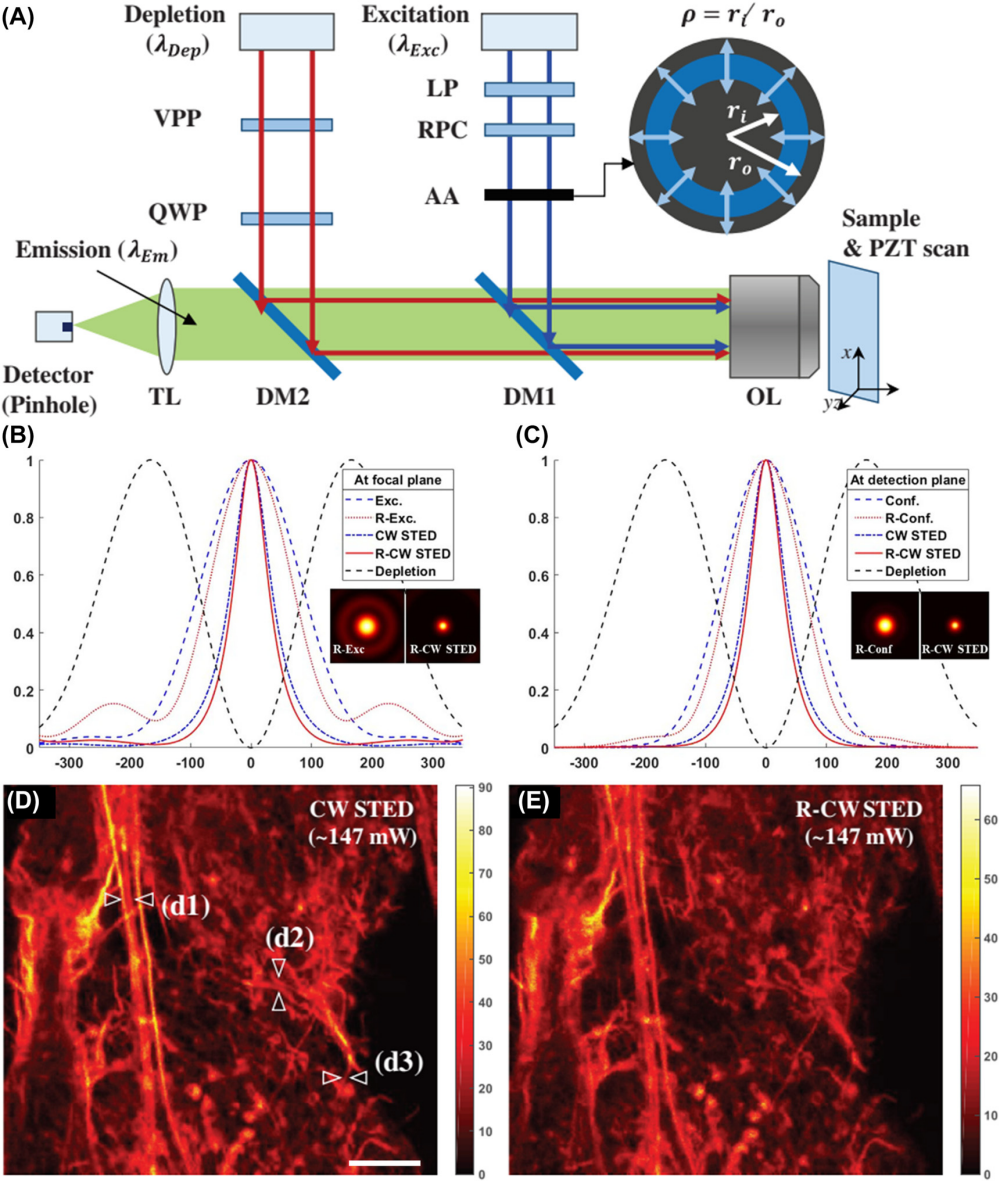
RVB-illuminated CW STED microscopy. (A) Sketch map of RVB-illuminated CW STED microscopy. (B) Normalized PSF functions on the focal plane with the conventional illumination (circularly polarized), RVB-illumination, CW STED, RVB-assisted CW STED and donut-shaped depletion illumination, respectively. (C) Normalized PSF functions on the detection plane in which the pinhole effect (0.66 AU) is considered for each case shown in (B). The parameter *ζ* was kept 5. *x*-axis units are nanometers. The inset is the corresponding intensity distribution of the focus in the case of RVB illumination and RVB-assisted CW STED. (D) Images of actin in NIH-3T3 cell with conventional CW STED. (E) Images of actin in NIH-3T3 cell using STED illuminated via annular RVB. The scale bar is 3 μm. Adapted from Ref. [118]. Copyright 2019 WILEY-VCH Verlag GmbH & Co. KGaA, Weinheim.

Lim et al. developed RVB-illuminated continuous wave (CW) STED microscopy, by which the lateral resolution can be enhanced about 12% by comparing with conventional CW STED [118]. Both theoretical and experimental analyses have been carried out to explore the possibility of resolution enhancement with annularly filtered RVB illumination. The sketch map of the RVB-illuminated CW STED microscopy is shown in Figure 10A, which indicates that the annular aperture has inner radius *r*_i_ and outer radius *r*_o_. Here, annular blocking ratio is defined as *ρ* = *r*_i_/*r*_o_, by varying which the amplitude modulation can be optimized so that the point spread function (PSF) or STED imaging performance can be optimized. Focal field near the focal plane of RVB can be written out based on vectorial diffraction theory. Taking the confocal effect of the pinhole and the STED effect by the donut-shaped depletion beam into consideration, the effect PSF can be calculated out. Through keeping depletion beam and the phosphor constant, the effective PSF of RVB-assisted CW STED and conventional CW STED can be calculated and compared. Here, *ζ* is used to describe the magnitude of the depletion PSF of the depletion beam. Figure 10B shows the calculated PSF functions on the focal plane with the conventional illumination (circular polarization), R-illumination, continuous wave (CW) stimulated emission depletion (STED), R-CW STED and donut-shaped depletion illumination, respectively. Figure 10C exhibits the normalized PSF functions for the detection plane in which the pinhole effect (0.66 Airy unit, AU) is considered for each case shown in Figure 10B. It can be seen from Figure 10B–C that the PSF was more condensed for RVB-assisted CW STED, and the FWHM of the effective PSF on the detection plane is smaller than that of conventional CW STED. In addition, undesired PSF sidelobes could be efficiently suppressed in the case of RVB illumination, as demonstrated by simulation results.

In experimental, conventional confocal microscope, CW STED, and RVB-assisted CW STED were used to imaging the same sample for in-situ comparing the imaging performance. Images of fluorescent beads, HaCaT cell and NIH-3T3 cell were obtained respectively by these three techniques. Figure 10D–E shows images of actin in NIH-3T3 cell obtained by CW STED and RVB-assisted CW STED, respectively. The comparison reveals obvious resolution-enhancement using RVB, which is consistent with the theoretical results. The resolution enhancement is reported to 12% compared to conventional CW STED.

This work [118] applied the annularly filtered RVB in STED for resolution enhancement with simple set-up in experimental and not only in theoretical, which is an advancement for STED, RVB application and super-resolution imaging, etc. But difficulties still exist in accurately adjusting the annular blocking ratio in practical.

#### 5.2.2 Subtraction imaging

Subtraction imaging possesses the improved resolution based on a simple subtraction of two images which are, respectively, acquired with closed and open pinhole [121, 122], or with different point spread functions (PSFs) [123], etc. Excess subtraction is a problem for subtraction imaging technique, leading to image degradation. The vector beam has been proposed to suppress the image degradation through eliminating the negative side lobe based on a new subtraction method, where the spatial resolution can up to 100 nm theoretically and experimentally [91, 138].

**Figure 11:**
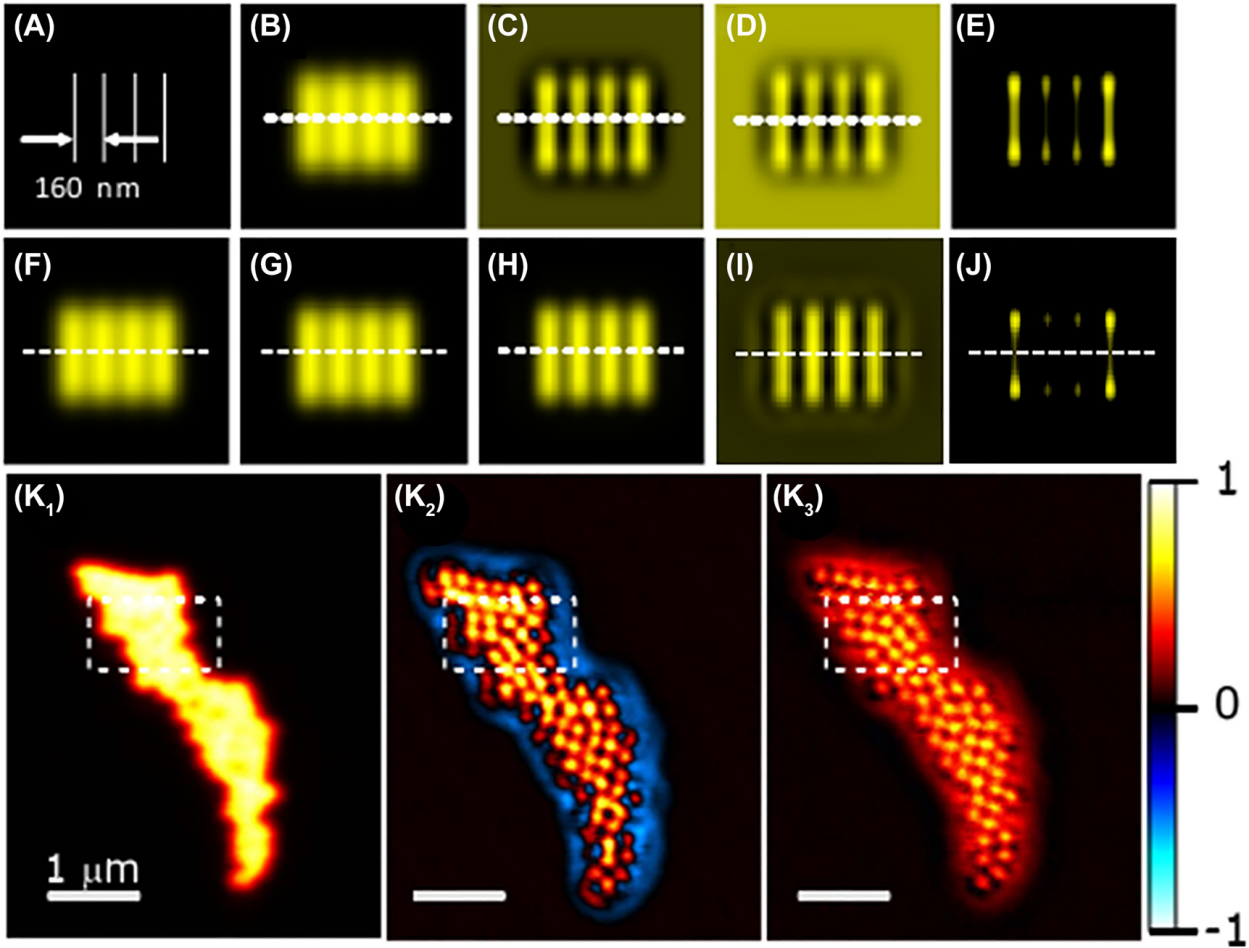
Subtracting imaging illuminated via Gaussian-AVB and RP-AVB. (A) Sketch map of the simulation object. (B–D) simulations of the subtraction imaging illuminated by Gaussian and AVB for *γ* = 0, *γ* = 0.25, and *γ* = 0.5, respectively. (E) Simulation image when the negative intensities resulted from the subtraction were set to zero in the case of *γ* = 0.5 under illumination of illuminated by Gaussian and AVB. (F) Simulation image illuminated by a circularly polarized Gaussian beam. (G) Simulations for the subtraction imaging in the case of Gaussian-AVB illumination, where *γ* was kept being 0.05. (H) Simulations for the subtraction imaging in the case of RV-AVB illumination, where *γ* was kept being 0.15. (I) Simulations for the subtraction imaging under illumination of flat-top and higher-order AVB, where *γ* was kept being 0.85. (J) Simulations for the subtraction imaging in the case of Gaussian-AVB illumination, where *γ* was kept being 0.85, and negative intensities were set to zero. Adapted from Ref. [138]. Copyright 2014 Optical Society of America. (K_1_) Images of aggregated fluorescent beads (200 nm in diameter) with a conventional confocal image illuminated by an LPB. (K_2_) Subtracted image illuminated via AP-LG_0,1_ and RP-LG_0,1_ mode superposition, and AP-LG_1,1_ mode. (K_3_) Subtracted image illuminated via AP-LG_1,1_ and RP-LG_1,1_ mode superposition, and AP-LG_2,1_ mode. Adapted from Ref. [91]. Copyright 2019 Optical Society of America.

Kozawa’s group has studied the superiority of vector beams for subtraction imaging recently. They theoretically demonstrated in 2014 that vector beams applied in subtraction imaging can obtain high spatial resolution and avoid the negative side lobe [138]. Imaging based on PSF subtraction was used to improve the resolution with advantages of simplifying calculation and instrumentation, where the PSFs under illumination of circularly polarized Gaussian beam, azimuthally polarized vector beam, and radially polarized vector beam was respectively calculated according to vectorial diffraction theory. The sketch map of the simulation object is shown in Figure 11A, and the corresponding simulation results are exhibited in Figure 11B–J. Subtracted images illuminated by Gaussian and AVB with subtraction coefficient *γ* = 0, *γ* = 0.25, and *γ* = 0.5 are shown as Figure 11B–D, respectively. Here, *γ* is defined by the equation below:

(14) $$PSF_{\text{sub}}=PSF_{\text{A}}-\gamma PSF_{\text{B}}$$

where PSF_sub_ refers to point spread functions corresponding to subtraction; PSF_A_ refers to point spread functions under A-typed beam illumination; PSF_B_ refers to point spread functions under B-typed beam illumination. Figure 11E is the simulation image when the negative intensities resulted from the subtraction were set to zero in the case of *γ* = 0.5 under illumination of illuminated by Gaussian and AVB. Simulation images under illumination of a circularly polarized Gaussian beam, and subtracted images in the case of Gaussian-AVB illumination where *γ* was kept being 0.05, and the case of RV-AVB illumination where *γ* was kept being 0.15 are also, respectively, simulated, as shown in Figure 11F–H. More importantly, subtracted images under illumination of flat-top (combination of radially and azimuthally polarized beam, named for its PSF has flat top) and higher-order AVB and under the Gaussian-APB illumination are respectively exhibited in Figure 11I and J, where *γ* was kept equaling 0.85, revealing that resolution can reach 100 nm under the illumination of flat-top-combination-beam which is consisted of radial and azimuthal polarizations.

A few years later, Kozawa’s group experimentally realized the spatial resolution of ∼100 nm using this method [91]. As shown in Figure 11K_1_–K_3_, aggregated yellow-green beads with diameter of 200 nm were, respectively, imaged under illumination of conventional linearly polarized beam, subtracted images illuminated via hybrid mode 1 (superposition of azimuthally polarized Laguerre–Gaussian (AP-LG_0,1_) and radially polarized Laguerre–Gaussian (RP-LG_0,1_ mode) and AP-LG_1,1_ mode, subtracted images illuminated via hybrid mode 2 (AP-LG_1,1_ and RP-LG_1,1_ mode superposition) and AP-LG_2,1_ mode, from which it can be seen that the combination method of hybrid mode 1 and AP-LG_1,1_ mode has obvious resolution improvement compared with the other combination method and the conventional method. Moreover, the combination method was verified to enhance resolution in biological imaging by imaging COS-7 cells labeled by anti-tubulin anti-bodies and AlexaFluor488-conjugated secondary antibodies.

These two works [91, 138] adopted two extraordinary-typed beams in subtraction imaging. One is superposition of AP-LG_0,1_ and RP-LG_0,1_ mode, and the other is AP-LG_1,1_ mode. The method can efficiently suppress the image degradation and improve the spatial resolution, but accurate subtraction coefficient is needed. In addition, the complexity will be increased for the superposition of AP-LG_0,1_ and RP-LG_01_ mode.

#### 5.2.3 Superoscillation imaging

Superoscillation imaging is a newly developed super-resolution technology based on superoscillation. In superoscillation a band-limited function locally oscillating faster than its fastest Fourier component under the condition that the superposition of its Fourier components is appropriately designed within a limited frequency range [191], [192], [193]. Superoscillation can generate a local “hot spot” in nanoscale, providing a new possible way to realize far-field super-resolution imaging.

Kozawa et al. proposed an approach of superoscillation-based superresolution imaging illuminated by RVB with a higher-order transverse mode under the condition of tight focusing [90]. Superoscillation criterion in tight focusing conditions was obtained based on vector diffraction theory for cylindrical vector beams because scalar theory is not precisely valid under tight focusing for that the influence of the vector nature of light cannot be ignored. According to the superoscillation criterion in tight focusing conditions, RP-LG_*p*,1_ beam was adopted to generate superoscillation focusing theoretically and experimentally. Simulation results reveal that lateral resolutions of confocal laser scanning microscopy (LSM) can reach 100 nm under RP-LG_3,1_ beam illumination.

**Figure 12:**
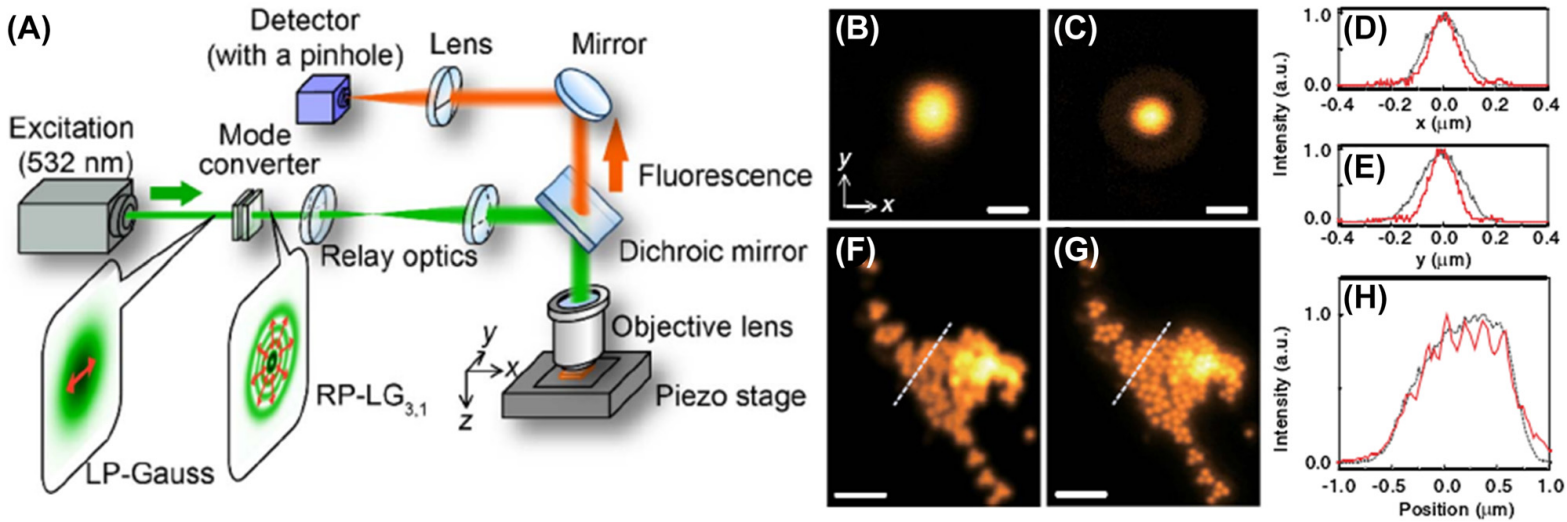
Superoscillation imaging under RP-LG_3,1_ illumination based on a confocal laser microscope. (A) Sketch map of superoscillation imaging system under RP-LG_3,1_ illumination. (B) Measured PSFs (0.5 AU) for LPB with superoscillation. The scale bar is 500 nm. (C) Measured PSFs (0.5 AU) for RP-LG_3;1_ beam with superoscillation. The scale bar is 500 nm. (D) Intensity profiles for the measured PSFs along the *x*-axes shown in (B) and (C). (E) Intensity profiles for the measured PSFs along *y*-axes shown in (B) and (C). (F) Image of clusters of fluorescent beads with diameters of 170 nm acquired under LPB (F) and RP-LG_3;1_ beam (G) illumination, respectively. The scale bars in (F, G) are both 1 μm. (G) Intensity profiles along the dashed lines in (F) and (G). The black dashed and red solid lines in (D), (E), and (H) correspond to LPB and RP-LG_3;1_ beam, respectively. Adapted from Ref. [90]. Copyright 2018 Optical Society of America under the terms of the OSA Open Access Publishing Agreement.

In experimental, as shown in Figure 12A, RP-LG_3,1_ beam was generated through converting a linearly polarized Gaussian beam to the target RP-LG_3,1_ mode via a mode converter consisting of two types of transmissive liquid crystal devices, where the laser wavelength was 532 nm, and the linearly polarized Gaussian beam was expanded in order for the central focusing spot of the converted RP-LG_3,1_ mode meeting the superoscillation criterion. A confocal laser microscope with a confocal pinhole (0.5 AU) in the image plane was developed to characterize the imaging performance in the case of superoscillation under RP-LG_3,1_ illumination. As shown in Figure 12B and C, the intensity distributions of the confocal PSFs respectively illuminated via LPB and RP-LG_3,1_ beam are obtained, where the imaging objects are 100 nm fluorescent beads. The corresponding intensity profiles along the *x*- and *y*-axes for each PSF are exhibited in Figure 12D and E, indicating that a smaller focusing spot can be obtained by RP-LG_3,1_ illumination compared with the case of LPB illumination. The images shown in Figure 12F and G shows reveal that the spatial resolution based on the superoscillation imaging illuminated via RP-LG_3,1_ mode is substantially higher than that obtained by LPB. The corresponding intensity profiles of the dashed lines in Figure 12F and G are exhibited in Figure 12H, which consistently illustrate the differences and superiority. Moreover, through imaging microtubules in fixed and labeled HeLa cells by LPB and RP-LG_3,1_ beam respectively, superoscillation spot generated under RP-LG_3,1_ beam illumination is proved to be useful for biological-sample imaging with high-resolution. The limitations of this method are the required relatively high input laser power for the weak intensity of the central spot under RP-LG_3,1_ beam illumination, and the degraded axial spatial resolution for the axial elongation of the focal spot.

## 6 Conclusion and perspectives

The development of optical field manipulation and generation of CVBs open up a new avenue for super-resolution imaging. This review is dedicated to presenting the recent progress in CVB-assisted super-resolution imaging, covering near-field and far-field microscopic techniques. The strategies for CVB-assisted super-resolution include excitation-volume decrease and excitation enhancement, which are mainly implemented by tightly focusing, plasmonic nanofocusing, depletion effect and polarization matching.

The typical CVB-based super-resolution imaging techniques in fields of both near-field and far-field microscopy are introduced, including tip-scanning imaging, nonlinear imaging, STED, subtraction imaging, and superoscillation imaging. The corresponding resolution enhancement factors are listed Table 1. The resolution enhancement factor is defined by *η* = (*R*_C_ − *R*_I_)/*R*_C_, where *R*_C_ refers to the resolution of control group; *R*_I_ refers to the resolution obtained by special light field illumination. It can be seen from Table 1 that CVBs show great potential for resolution improvement of super-resolution imaging, as well as orientation information acquirement, signal-to-noise enhancement, efficiency increase, etc. CVBs also have nonnegligible disadvantages that excitation-volume decrease and excitation enhancement are often performed in near-field regions, making it impractical to image samples on large scale. To date, although the principles and underlying physics of CVB-assisted resolution enhancement have been individually stated in many works, it is still challenging to provide a general rule and quantitative statement of the improving performance in different imaging systems. Systematic theories are highly demanded in the future. It is promising to develop novel techniques with ultra-high resolution using CVBs as illumination source, which contributes to the fields of nanophotonics, nanobiology, nanomaterials, etc.

**Table 1:** Comparison of the typical super-resolution optical microscopy illuminated via CVBs.

Imaging techniques		Illumination light	Control group	Resolution enhancement	Other superiorities and shortages
		field and resolution *R*_I_	and resolution *R*_C_	factors *η*	
Tip scanning imaging	Using gold-coated SiO_2_ tip with nanoscale annular slits [44]	RVB, ∼10 nm [44]	LPB illumination, commercial aperture-type tip, ∼60 nm [44]	∼83%	High signal-to-noise ratio of ∼18.2 [44]; expensive preparation process
	Using fibre-coupled nanowire probe [92]	RVB, ∼1 nm [92]	Commercial TERS system [194], ∼10 nm	∼90%	Broad working bandwidth and high external nanofocusing efficiency of ∼50% [92]; difficult to possess high stability and repeatability
Nonlinear imaging	CARS combined with tip-enhanced microscopy [153]	RVB, ∼80 nm for mitochondrion [153]	AFM, unable to distinguish the ∼80 nm sized hole in the mitochondrion [153]	∼73%, compared to the resolution of ∼300 nm for conventional CARS [195]	Enhanced CARS signal of 6-fold compared to LPB and improved signal-to-background ratio of ∼3 times compared to the LPB [153]; complex optical system; difficult to realize the alignment of tip axis, pump beam and Stokes beam
SHG microscopy [154]	RVB, vertically oriented GaAs nanowires with 40 nm diameter can be distinguished [154]	LPB and AVB illumination, unable to distinguish the vertically oriented GaAs nanowires with 40 nm diameter [154]	–	Enhanced SHG intensity of 3–5 folds and 30 folds respectively obtained by experiment and simulations compared to LPB
STED microscopy	Total internal reflection STED	Use RVB as excitation beam, a lateral resolution of ∼50 nm and an axial confinement ∼70 nm [45]	Conventional STED with sacrificed axial resolution; 3D STED a lateral resolution of 50 nm and an axial resolution of 150 nm [196]	∼53% in axial	Potential to reduce photo-bleaching of fluorophores and phototoxicity to living samples [45]; slightly reduced lateral resolution compared to conventional STED with resolution of 35 nm [128]
CW STED	RVB with energy modulation by an annular aperture, used as excitation beam [118]	Conventional CW STED	12% [118]	Simple set-up; difficult to adjust the annular blocking ratio in practical
Subtraction imaging		Combination of radially and azimuthally polarized beam, ∼100 nm theoretically and experimentally [91,138]	LPB illumination, indistinguishable fluorescent beads with 200 nm diameter [91]	>50%	Suppressing the image degradation; increased complexity for the superposition of AP-LG_0,1_ and RP-LG_01_ mode
Superoscillation imaging		RP-LG_3,1_ beam, ∼100 nm theoretically and ∼100 nm experimentally [90]	LPB illumination, indistinguishable fluorescent beads with 170 nm diameter [90]	>41%	Lower peak intensity of the superoscillation spot compared to LPB and degraded axial spatial resolution [90]
Resolution enhancement factors *η* = (*R*_C_ − *R*_I_)/*R*_C_					
